# Evaluation of Psychological and Physical Violence towards Children and Adolescents before and during the COVID-19 Pandemic in the Lodz Voivodship

**DOI:** 10.3390/brainsci14010024

**Published:** 2023-12-25

**Authors:** Jagoda Grzejszczak, Agata Gabryelska, Magdalena Kotlicka-Antczak, Dominik Strzelecki

**Affiliations:** 1 Department of Child and Adolescent Psychiatry, Medical University of Lodz, 92-216 Lodz, Poland; magdalena.kotlicka-antczak@umed.lodz.pl; 2 Department of Sleep Medicine and Metabolic Disorders, Medical University of Lodz, 92-215 Lodz, Poland; agata.gabryelska@gmail.com; 3 Department of Affective and Psychotic Disorders, Medical University of Lodz, 92-216 Lodz, Poland; dominik.strzelecki@umed.lodz.pl

**Keywords:** COVID-19, pandemic, child, adolescent, physical abuse, psychological maltreatment

## Abstract

Background: It has been shown that the course of COVID-19 infection in the under-18 population was in many cases sparsely symptomatic. In contrast, the impact of the pandemic on the psychological state is quite different. The risk of psychopathological symptoms in children and adolescents increased and the course of already present psychiatric disorders has often been exacerbated. Objectives: Thus, this study aimed to evaluate the prevalence of psychological and physical violence among children and adolescents and its change during the COVID-19 pandemic, as well as to investigate various factors that might affect violence. Methods: In this survey study, 782 responses were included, with 480 collected during the second and 302 during the fourth wave of COVID-19. In this cross-sectional study, an anonymous questionnaire was used to collect demographic data, medical history, mental state, psychopathological symptoms, as well as the presence of psychological, physical violence, and suicidal self-harm behaviors before (retrospectively) and during the COVID-19 pandemic in the under-18 population of the Lodz Voivodship. The survey was prepared using Google Forms. Results: A decrease in the prevalence of physical violence during both waves of the pandemic has been observed (6.39% vs. 3.45%; *p* < 0.001), with only a similar trend present for psychological violence 16.75% vs. 14.71%; *p* = 0.081). No difference between physical and psychological violence was present in different pandemic waves, type of flat or house individuals lived in, availability of one’s room, number of people living in the house, number of siblings, and type of school classes (*p* > 0.050). Older children (>15 years old) were more likely to be victims of psychological violence before and during the pandemic (both *p* < 0.001). A statistically significant model was obtained for psychological violence before (*p* < 0.001, R^2^ = 0.011) and during the pandemic (*p* = 0.007, R^2^ = 0.032). Risk factors for psychological violence before the pandemic included male gender (B = 0.531, *p* = 0.009, OR = 1.700), older age (B = 0.869, *p* = 0.001, OR = 2.385), and smaller city size (B = −0.187, *p* = 0.004, OR = 0.829), while for psychological violence during the pandemic, the risk factors were only male gender (B = 0.482, *p* = 0.022, OR = 1.620) and older age (B = 0.555, *p* = 0.046, OR = 1.742). No statistically significant models were created for physical violence (*p* > 0.050). Conclusions: The observed decrease in physical violence during the COVID-19 pandemic suggests that in the studied group, home environment was not the main source of physical violence. Yet, we did not find any predicting factors for this form of violence. Violence, both physical and psychological, is a dangerous phenomenon in the under-18 population both in the pre-pandemic period and in crisis situations such as the pandemic.

## 1. Introduction

Violence is a serious problem in the population of children and adolescents, while family and school are among the most common sources of violence in the under-18 population [1]. In terms of the Convention on the Rights of the Child, a child means “every human being below the age of eighteen years unless under the law applicable to the child, majority is attained earlier” [2]. Among the most common sources of violence in the under-18 population is the family [3]. Analyzing the problem in a cultural context seems appropriate [4]. Violence is defined by the World Health Organization (WHO) as “the intentional use of physical force or power, threatened or actual, against oneself, another person, or against a group or community, that either results in or has a high likelihood of resulting in injury, death, psychological harm, maldevelopment or deprivation” [5]. The definition according to the WHO distinguishes an additional subgroup of children in adolescence [6]. According to this, adolescence as the second decade of life (10–19 years of age) is a time when significant physical, psychological, and social changes occur [7]. In Europe, almost every third child experiences some form of violence, according to the World Health Organization [8]. As a European and largely conservative country in terms of worldview, Poles attach great importance to tradition and classical educational models are still common, but we are more liberal than a few years ago [9]. We are also a country with moderately favorable economic conditions [10]. A number of risk factors for violence are typical regardless of cultural context: young age, male gender, weak social and family ties, separation from parents, peer environment, virtual violence, and low economic status of the family, but also intergenerational cultural transmission of violent behaviors [1,7]. The time of the COVID-19 pandemic in Poland, but also in most countries of the world, was associated with several burdensome circumstances [11], with domestic isolation, reduced social contact, worse economic conditions, and stress related to the risk of the disease to name a few [12]. Since violence is a very broad concept [13], we distinguish between psychological (inadequate control, criticism, restriction of contact, humiliation, etc.), physical (hitting, choking, jerking, maiming), as well as sexual violence, neglect, or violence limited to the Internet environment [11,14]. The pandemic period also in Poland was associated with a long period of home isolation for epidemiological reasons [13,15,16]. Home isolation was associated with more time spent with family members against the simultaneous backdrop of uncertainty of economic and business events, the risk of contracting the virus, but also a reduction in social contact, the need for many people to function in a small space, and, at the same time, increased stress levels [17,18]. Moreover, adults who do not provide adequate care to minors expose them to extensive emotional and social consequences and an increased risk of being a victim of future violence [19]. Therefore, the study aimed to evaluate the changes in the frequency of psychological and physical violence toward children and adolescents before and during the pandemic, as well as to assess various factors affecting its prevalence and possible changes. The hypothesis of the study was that the severity of physical and psychological violence among residents of Lodz province under 18 during the COVID-19 pandemic would be reduced based on the protective factor of strong and supportive relationships in the home environment, on which daily functioning was based in the era of domestic epidemiological isolation during the second and fourth waves of the pandemic in Poland. Another hypothesis was that there would be a reduction in the severity of violence during the era of home isolation due to a reduction in forms of violence stemming from the school environment.

## 2. Materials and Methods

### 2.1. Study Design and Sample

The study included the Polish population of children and adolescents of the Lodz Voivodeship, Poland, during the second wave (2021) and the fourth wave (2022) of the COVID-19 pandemic. It should be mentioned that in Polish schools, during the second wave of the pandemic, learning took place exclusively online, while during the fourth wave, classes were held in a mixed form. The age of the respondents covered the population range of 6–18 years, which is the same as the age of children covered by compulsory education in Poland. The self-administered anonymous online questionnaire was available via a Google spreadsheet online. The study has a cross-sectional character, including the retrospective character of answers concerning the time period before the COVID-19 pandemic. The questions contained in the survey concerned both the current situation and, retrospectively, the situation preceding the pandemics. This is a limitation of the study as it does not have an observational form.

Participants were invited to take the survey through individual contact with the directors of educational institutions in the Lodz area. Of the 3383 registered institutions of this type, 86 schools were invited to the survey. Four of them refused to take part in our project. Each principal received the content of the questionnaire’s questions for review and discussion with the school’s psychological and pedagogical team before the questionnaires were distributed to parents via e-mail. These, after agreeing to their child’s participation in the study, passed them on to their children. All participants were aware of the study conditions and gave informed consent to participate. Confidentiality and anonymity were maintained, and no data that could help to identify a responder were collected. In total, 823 responses were collected. From the analysis, 41 individuals were excluded (1 due to a wrong date of birth and 40 due to not fulfilling the age criteria for inclusion—over 18 years old). Further, since only 2 participants responded that they lived in the capital, Warsaw, during the pandemic period, they were added to a group who lived in a city of 100–1000 thousand people together, in effect making a group living in a city with an over 100 thousand population.

### 2.2. Measurement Tools

The survey was prepared using Google Forms (see Supplementary Materials for Polish language version of the questionnaire). The self-administered anonymous online questionnaire was available via a Google spreadsheet online. Questions about experienced violent behavior were prepared based on the Early Trauma Inventory (J.D. Bremner 2000, polish translation and adaptation under the supervision of B. Spila). The original questionnaire was divided into demographic data about the participant, household, and family members, general well-being during the pandemic, the mental state during the pandemic, the prevalence of COVID-19 infection concerns, need to maintain social contact during the pandemic, coping at school, psychopathological symptoms before and during the pandemic (anxiety, depressive symptoms), physical violence, psychological violence, suicidal behavior, psychoactive substance use, and physical activity. The questions referred to the 2 time points—first, during the pandemic (present moment while filling out the questionnaire) and second, before the pandemic (answered retrospectively by participants)—thus, the study did not have an observatory design. To defrieciente between these 2 time points within the article, they are referred to as during the pandemic or before/pre-pandemic period. The questionnaire was not validated. The purpose of the survey was to examine the occurrence/severity of symptoms, and the aim was to determine the scale of difficulty, not to directly make a diagnosis, which is not the purpose of a directly validated tool. We also do not have a directly validated tool that would cover such a broad context of the problem as we could capture with our own tool.

### 2.3. Statistical Analysis

Statistical analysis was performed at a significance level of 0.05 using two-tailed tests. The normality of the distribution of variables was tested with the Shapiro–Wilk test. For variables with a normal distribution, the data are presented as the mean with the standard deviation; for ordinal variables, the data are shown as frequencies and the number of cases; for variables with a distribution other than normal, the data are presented as the median with the interquartile range (IQR). Chi-square, Chi-square tests with Yate’s correction, and Fisher’s tests were used to assess nominal variables in situations where the size of the smallest group was, respectively, above 15, in the range 5–15, and below 5. Comparisons of 2 independent groups were made using Student’s *t*-test (for variables with a normal distribution) and the Mann–Whitney U test (for variables with a different distribution than normal). More than 2 independent groups were compared using the Kruskal–Wallis test (comparisons of multiple groups were only performed on parameters with distributions other than normal) with a Dunn–Bonferonni test for post hoc assessment. Dependent groups were compared with Student’s *t*-test for dependent variables (for variables with a normal distribution) or Wilcoxon (for variables with a different distribution than normal). Multinomial logistic regression was used to analyze the effect of chosen variables on the presence of violence. The analysis was performed using SPSS Statistics version 28 (IBM, Armonk, NY, USA).

## 3. Results

**Figure 1:**
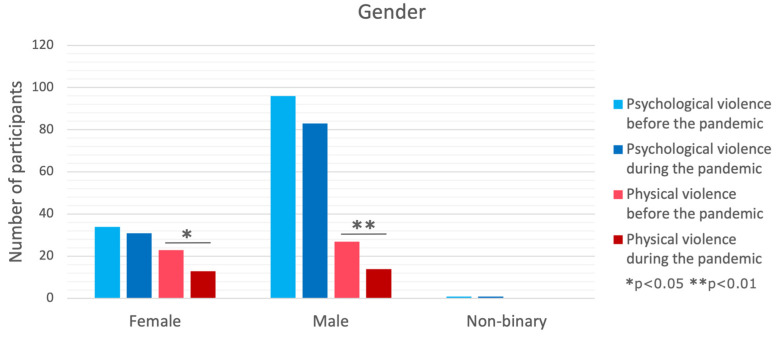
Comparison of violence forms in the context of gender.

**Figure 2:**
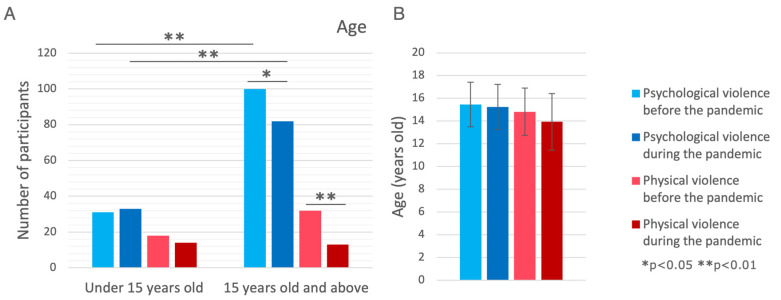
Comparison of violence forms in the context of age. (**A**) Shown as number of subjects. (**B**) Shown as mean ± standard deviation.

**Figure 3:**
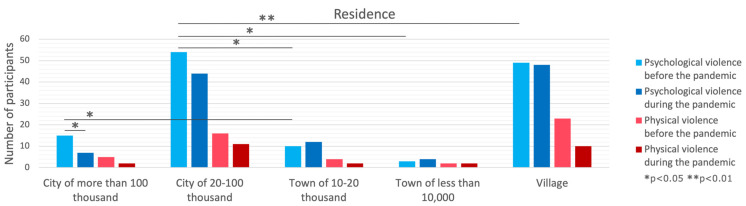
Comparison of violence forms in the context of residence.

**Figure 4:**
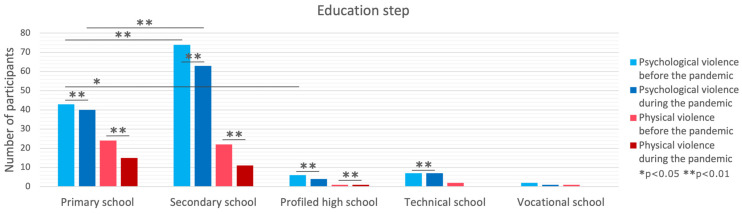
Comparison of violence forms in the context of educational level.

In the final analysis, 782 responses were included: 480 responses were collected during the second wave of COVID-19, and 302 during the fourth wave. The general characteristics of the participants are shown in Table 1 and Figure 1, Figure 2, Figure 3 and Figure 4 (detailed data are provided in Supplementary Table S1).

A decrease in the prevalence of physical violence before and during the pandemic has been observed (6.39% vs. 3.45%; *p* < 0.001), while only a tendency in the same direction was shown for psychological violence, without statistical significance (16.75% vs. 14.71%; *p* = 0.081).

No difference between physical and psychological violence was present in different pandemic waves, type of flat or house individuals lived in, availability of one’s room, number of people living in the house, number of siblings, and type of school classes.

### 3.1. Gender

No differences between genders were observed in physical violence before and during the pandemic, while a significant difference was present in psychological violence before (*p* = 0.003) and during (*p* = 0.031) the pandemic. The post hoc tests showed that in both cases, the prevalence was higher among non-binary participants compared to girls (before *p* = 0.003, during *p* = 0.002). Furthermore, no changes were observed in psychological violence frequency before and during the pandemic. While for physical violence, a decrease was observed among girls (*p* = 0.018) and boys (*p* = 0.009). Yet, no difference in the decrease between genders was present (*p* = 0.888).

### 3.2. Age

Older children (>15 years old) were more likely to be victims of psychological violence before and during the pandemic (both *p* < 0.001). In children below 15 years old, no changes in physical and psychological violence were present before and during the pandemic (*p* > 0.05), while in the group aged 15 years old and older, a decrease in psychological (0 = 0.018) and physical (*p* < 0.001) violence was observed during the pandemic when compared to the pre-pandemic period. However, no statistically significant difference between these age groups with a change in psychological (0 = 0.057) and physical (*p* = 0.097) violence was achieved.

### 3.3. Residence

No differences between city sizes were observed in physical violence frequency before and during the pandemic, while a significant difference was present in psychological violence only before (*p* = 0.026) but not during (*p* = 0.394) the pandemic. The post hoc tests showed that in the pre-pandemic period in a city with a size of 20–100 thousand, the prevalence of psychological violence was higher compared to a city size of 10–20 thousand (*p* = 0.027), a city below 10 thousand (*p* = 0.026), or villages (*p* = 0.006). Similarly, cities over 100 thousand had a greater frequency of psychological violence than cities below 10 thousand (*p* = 0.042). Further, no change was present in the frequency of physical violence before and during the pandemic. Moreover, a decrease in psychological violence was observed only in cities over 100 thousand (*p* = 0.021), while a tendency was present for city sizes of 20–100 thousand (*p* = 0.068); for smaller cities, no change was observed. A difference in the decrease in psychological violence between city sizes was observed (*p* = 0.026). The post hoc tests showed that the decrease in psychological violence was greater in a city over 100 thousand compared to a city size of 20–100 thousand (*p* = 0.006), between 10 and 20 thousand (*p* = 0.023), or below 10 thousand (*p* = 0.006), while compared to the city size of 20–100 thousand, only a tendency was present (*p* = 0.068).

### 3.4. Education Level

No differences between education levels were observed in physical violence before and during the pandemic, while a significant difference was present in psychological violence before (*p* < 0.001) and during (*p* = 0.039) the pandemic. The post hoc tests showed that in the pre-pandemic period, the prevalence of psychological violence was lower in primary school children compared to secondary (*p* < 0.001) and profiled high school (*p* = 0.021) children, while during the pandemic, the level of psychological violence was lower in primary school children only in comparison to secondary school children (*p* = 0.001). Furthermore, a significant decrease was observed in physical violence from before to during the pandemic in primary and secondary school children, as well as profiled high school children (all *p* < 0.001), while for psychological violence, the decrease was observed in all groups (*p* < 0.001) except vocational school children (*p* = 0.361). Yet, no difference in the decrease between educational levels was present for either physical (*p* = 0.503) or psychological violence (*p* = 0.357).

### 3.5. Multinominal Logistic Regression

**Figure 5:**
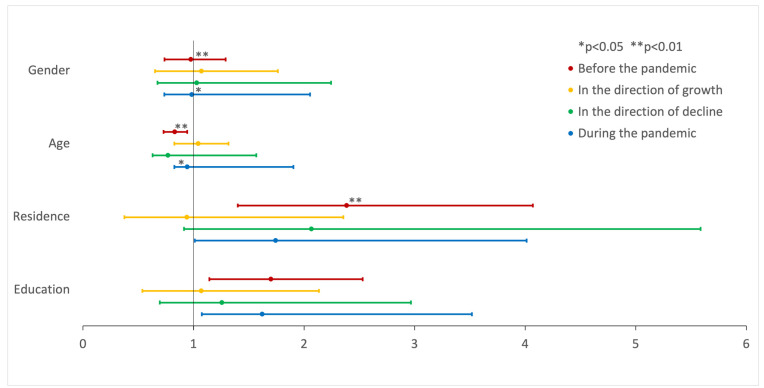
Summary of odds ratio for psychological violence. Odds ratio with 95% confidence intervals of evaluated parameters as risk factors for the presence or change in psychological violence in chosen time periods.

**Figure 6:**
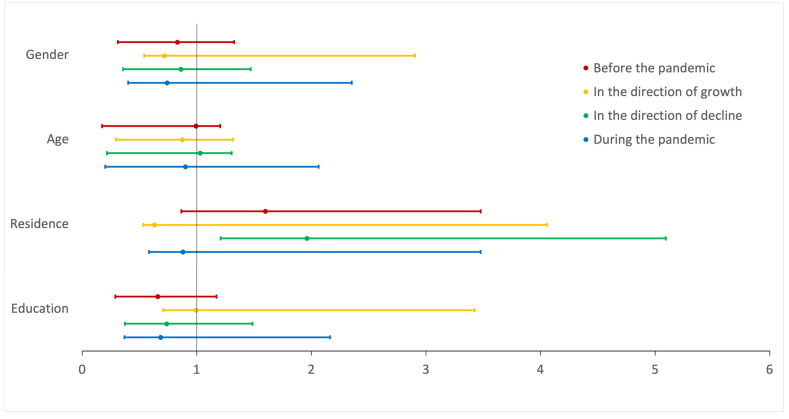
Summary of odds ratio for physical violence. Odds ratio with 95% confidence intervals of evaluated parameters as risk factors for the presence or change in physical violence in chosen time periods.

The models were prepared to assess the risk factors for psychological and physical violence before and during the pandemic, as well as for the increase or decrease in violence. A statistically significant model was obtained for psychological violence before (*p* < 0.001, R^2^ = 0.011) and during (*p* = 0.007, R^2^ = 0.032) the pandemic. Risk factors for psychological violence before the pandemic included male gender (B = 0.531, *p* = 0.009, OR = 1.700; Figure 5), older age (B = 0.869, *p* = 0.001, OR = 2.385; Figure 5), and smaller city size (B = −0.187, *p* = 0.004, OR = 0.829; Figure 5), while for psychological violence during the pandemic, the risk factors were only male gender (B = 0.482, *p* = 0.022, OR = 1.620; Figure 5) and older age (B = 0.555, *p* = 0.046, OR = 1.742; Figure 5). No statistically significant risk factors for physical violence before and during the pandemic were identified (Figure 6). Full data for the created models are presented in Table 2.

## 4. Discussion

Violence is a very complex phenomenon with serious consequences [1]. The overall prevalence of violence against children in COVID-19 was estimated at 39%, with a prevalence of 18% in Europe, 35% in Asia, 47% in North America, and 88% in Africa, respectively [2]. However, this is not confirmed by our research, which shows a decrease in the prevalence of physical violence before and during the pandemic, while only a tendency in the same direction was shown for psychological violence, without statistical significance. Only a few studies show a decrease in the incidence of physical or psychological violence during the COVID-19 pandemic compared to the pre-pandemic period. It is important to emphasize, however, that most of the research conducted to assess the scale of the problem of violence in this period was conducted on a population of children hospitalized or seeking support on helplines, which might contribute to higher prevalence rates [3,4,5]. This does not provide a cross-sectional study group. Moreover, the significant role of parents’ support in the social field and the importance of psychoeducation related to behavioral influences are emphasized as important protective factors in the context of violence against children [6,7]. The recently published findings of German researchers seem interesting. They, like us, analyze the problem of violence against children from the perspective of the family system, proving that some forms of violence, including violence against children, have decreased during the pandemic. They point out that it is impossible to reliably compare the results with those from before the pandemic due to the lack of precise data in a broad context [8]. Analysis of risk factors for this phenomenon aims to enable a better understanding of related processes, as well as more efficient prevention [9]. In our project, we investigated parameters of psychological well-being (including the presence of psychological and physical violence) in the population of the Lodz province aged up to 18 years before and during the COVID-19 pandemic. The pandemic period was inextricably linked to home isolation, and thus remote learning in many Polish schools, but also more frequent and prolonged contact with family members and other household members [10]. There was a general increase in the prevalence of violence during the COVID-19 pandemic period in the population we studied, as reported by other researchers [11]. Increased time spent in isolation with perpetrators of violence coming from the home environment, as well as limited accessibility to supportive institutions (psychologists, school, church, peers), greater emphasis on online forms of contact (an increase in cyber-bullying among students), and the global economic crisis had an indirect effect on the increase in domestic conflicts [12]. In our study, on the other hand, there was a marked decrease in the frequency of physical violence compared to the pre-pandemic period. We also observed a trend toward a decrease in the frequency of psychological violence.

Analysis of gender as a risk factor showed a marked decrease in the percentage of boys experiencing psychological violence during the pandemic period (with teaching mostly at home), with no significant change in the prevalence of psychological violence among both girls and declared non-binary students. The higher prevalence of both types of violence both before and during the pandemic in boys has been previously reported [14]. Statistics indicate that boys are more frequently involved in violence and bullying that occurs in the school environment [13]. The greatest increase in psychological violence has been reported among students over the age of 15, which may be related to difficulties in family communication among adolescents. It has also been shown that the pandemic period increased overall family stress levels, which correlated with an increase in psychological violence at home [15]. There is also a lack of a real and adapted policy to reduce the incidence of addiction to alcohol and other psychoactive substances at home, which significantly affects the level of aggression present there. The data collected so far suggest a significant increase in people suspected of alcohol addiction related to home isolation, lack of adequate support, or feelings of loneliness related to the pandemic [16]. The availability of various forms of therapy, including systemic interventions, worsened during the pandemic period [17,18]. The reported decline in the incidence of physical and psychological violence across the age population underscores the source of this risk in peer interactions [19]. On the one hand, violent behavior of a diverse nature can occur at school, but on the other hand, school is (and certainly should be) an important place for both learning behavior by observation, as well as providing opportunities to implement multidirectional preventive measures [20]. It has been proven that teaching stress-coping strategies in elementary schools translates into less aggressive behavior in the older grades [21]. It seems that in Polish reality, the emphasis placed on this issue is too low and the school is also not prepared, as well as sufficiently supported, to organize activities to prevent violent behavior, as well as to solve these types of problems if they occur, which is confirmed by quite numerous media reports. Intolerant and aggressive statements made by right-wing politicians on topics such as people identifying as LGBTQ+ in public media are also a significant problem.

Our results seem to confirm that school can, like other off-site interactions, be an important source of the occurrence of violence, both physical and psychological, and that home isolation and limiting contact outside the home have been shown to have a protective effect [22]. In contrast, the lack of significant change in the severity of psychological violence in the female population may indicate that less frequent out-of-home interaction does not reduce (or increase) exposure to violence. This may suggest that interactions outside the home environment are less important to the incidence of violent behavior against women. This may be directly related to the patriarchal paradigm still prevalent in Polish families and, unfortunately, the quite common occurrence of violence against women [23,24].

The data collected in the surveys also indicated a disturbing problem for non-binary people. Two-thirds of respondents in this group experienced psychological violence both before and during the pandemic, all experiencing physical violence. This confirms the findings of Swedish researchers, who noted an increased risk of violence against non-binary youth in relation to male or female peers and the above-mentioned activity of some politicians [25]. The problem seems relevant in a Polish society with a conservative educational model [26]. Capturing violence in the context of nonbinary gender in the era of the COVID-19 pandemic, however, has been poorly studied to date.

The reported decline in both psychological and physical violence in large cities and their concomitant increase in smaller towns and rural areas may suggest greater accessibility to both therapeutic environments and greater capacity to build support networks in larger urban centers or metropolitan areas [27]. Regardless of the pandemic period, studies indicate a higher incidence of violence in rural areas compared to cities [28]. A characteristic phenomenon in small-town and especially rural areas is multi-generational housing, which is assumed to increase the number of potential perpetrators of domestic violence, but also broadens the range of people who can provide support [29]. The increase in the incidence of pre-pandemic violence among students in various educational institutions with lower levels of general (vocational) education shows that the phenomenon of violence is influenced by both economic status and educational level [30]. A better quality of education reduced the risk of physical and psychological violence during the pandemic. In contrast, we did not prove the effect of economic status on the phenomenon of violence (room sharing, family fertility), as mentioned in earlier studies [31].

Reference should also be made to global working papers, technical reports, and journal articles on the impact of COVID-19 on violence against children. The data contained in them are not entirely consistent and, at the same time, not entirely identical to the results we obtained [32]. The studies mainly contain data on domestic violence (physical and psychological), while not including educational institutions or non-profits as protective factors, which at the same time have a major role in prevention [33]. Although all studies were interested in understanding whether such violence has increased during the pandemic, a few also examined the specific stressors that can lead to violence [34]. What is important, therefore, is the stressor itself in the form of a pandemic, and a good enough family system is itself a protective factor, so this does not confirm its direct contribution to the increase in the phenomenon of violence. In addition, most of the studies relied on administrative data, leaving out tools for obtaining direct information, which anonymous surveys undoubtedly do [35]. The different types of research designs and the lack of standardized research tools do not allow adequate comparison of the data collected. Mixed data were obtained regarding the use of at least a helpline in the denunciation of perpetrators of violence, which may indicate the varying degree of involvement of the children’s school environment, mental health workers, or public officials in the violence prevention process, depending on the specific facility and, more specifically, the staff employed there [36,37,38,39]. The phenomenon of violence is etiologically diverse, which is also confirmed by our study during the COVID-19 pandemic.

It is important to mention the limitations of the survey conducted. Because the survey was conducted online, it is difficult to verify the respondents and their affiliation with the target group. However, it was not a publicly available form but sent to parents of students from specific educational institutions, which limits the aforementioned inadequacy of responses. Direct access to the questionnaire by parents could theoretically limit access to the questionnaire by children from violent families due to the deliberate concealment of the phenomenon occurring at home despite the anonymity of the questionnaire. Unfortunately, however, regulations in Poland do not allow conducting any research (even surveys) in the population under 18 years of age without the consent of a parent, which we are unable to omit in any research project. Another limitation was the lack of the use of validated questionnaires to study psychopathological symptoms. However, the questionnaire that was prepared was comprehensive and tailored to the newly emerging crisis of the COVID-19 pandemic, its course, limitations, and effects. Last but not least, it has to be stated that the design of the study itself is burdened as it is not a prospective observational study. Participants during the pandemic answered questions regarding the present time (COVID-19 pandemic) and were asked to assess the situation before the pandemic retrospectively. Therefore, the retrospective answers could be influenced by time that passed from individuals’ experiences, so the answers could be both diminished or exaggerated; thus, the results obtained in the study should be interpreted with caution, taking into consideration these limitations of the study design.

## 5. Conclusions

The observed decrease in physical violence during the COVID-19 pandemic suggests that in the studied group, home environment was not the main source of physical violence, possibly pointing to peer groups and educational settings as the main sources of this form of violence. However, it is important to point out that we did not find any predicting factors for physical violence. While the frequency of physiological violence did not change, its prevalence was much greater compared to physical violence. Violence, both physical and psychological, is a dangerous phenomenon in the under-18 population both in the pre-pandemic period and in crisis situations, such as the pandemic, which highlights the need for not only better forms of prevention to reduce the incidence of violence among children and adolescents but also for educating adults closest to this population, making them more equipped in recognizing psychological and physical violence in this age group.

## Figures and Tables

**Table 1:** Baseline characteristics of the study group.

		Whole Study Group	Psychological Violence		Physical Violence	
			Before the Pandemic	During the Pandemic	Before the Pandemic	Before the Pandemic
Whole study group		782	131 (16.8%)	115 (14.7%)	50 (6.4%)	27 (3.5%)
Pandemic wave	2^nd^	480 (61.4%)	79 (16.5%)	70 (14.6%)	26 (5.4%)	15 (3.1%)
	4^th^	302 (38.6%)	52 (17.2%)	45 (14.9%)	24 (7.9%)	12 (4.0%)
Building	Block of flats	197 (25.2%)	32 (16.2%)	26 (13.2%)	14 (7.1%)	7 (3.6%)
	Terraced house	39 (5.0%)	7 (17.9%)	8 (20.5%)	1 (2.6%)	0 (0.0%)
	Detached house	546 (69.8%)	92 (16.8%)	81 (14.8%)	35 (6.4%)	20 (3.7%)
Room	Own room	15 (1.9%)	4 (26.7%)	3 (20%)	2 (13.3%)	1 (6.7%)
	Shared with someone	111 (14.2%)	20 (18%)	16 (14.4%)	8 (7.2%)	4 (3.6%)
	no own	656 (83.9%)	107 (16.3%)	96 (14.6%)	40 (6.1%)	22 (3.4%)
Number of household members	2	49 (6.3%)	8 (16.3%)	7 (14.3%)	3 (6.1%)	2 (4.1%)
	3	185 (23.7%)	39 (21.1%)	30 (16.2%)	16 (8.6%)	12 (6.5%)
	4	317 (40.5%)	49 (15.5%)	45 (14.2%)	17 (5.4%)	9 (2.8%)
	5	145 (18.5%)	26 (17.9%)	25 (17.2%)	11 (7.6%)	4 (2.8%)
	6 and more	86 (11.0%)	9 (10.5%)	8 (9.3%)	3 (3.5%)	0 (0.%)
Siblings	0	154 (19.7%)	27 (17.5%)	18 (11.7%)	9 (5.8%)	3 (1.9%)
	1	431 (55.1%)	75 (17.4%)	65 (15.1%)	26 (6%)	15 (3.5%)
	2	142 (18.2%)	22 (15.4%)	22 (15.4%)	11 (7.7%)	7 (4.9%)
	3	38 (4.8%)	6 (15.8%)	7 (18.4%)	3 (7.9%)	1 (2.6%)
	4 and more	17 (2.2%)	1 (5.9%)	3 (17.6%)	1 (5.9%)	1 (5.9%)
Type of school lessons	Stationary	42 (5.4%)	3 (7.1%)	4 (9.5%)	5 (11.9%)	3 (7.1%)
	Remote	707 (90.4%)	121 (17.1%)	108 (15.3%)	42 (5.9%)	21 (3%)
	Hybrid	33 (4.2%)	7 (21.2%)	3 (9.1%)	3 (9.1%)	3 (9.1%)

The results for continuous variables are shown as mean ± standard deviation, while for categorical variables as a number of subjects (percentage of the group).

**Table 2:** Multinominal logistic regression model assessing risk factor of violence.

	Psychological Violence			
	Before the Pandemic	Change		During the Pandemic
		In the Direction of Growth	In the Direction of Decline	
Model	*p* < 0.001, R^2^ = 0.011	*p* = 0.076		*p* = 0.007, R^2^ = 0.032
Intercept	B = −1.903, *p* < 0.001	B = −3.226, *p* < 0.001	B = −2.454, *p* < 0.001	B = −2.232, *p* < 0.001
Gender	B = 0.531, *p* = 0.009, OR = 1.700 (95%CI 1.143–2.529)	B = 0.068, *p* = 0.848, OR = 1.070 (95%CI 0.536–2.134)	B = 0.228, *p* = 0.451, OR = 1.256 (95%CI 0.695–2.271)	B = 0.482, *p* = 0.022, OR = 1.620 (95%CI 1.074–2.443)
Age	B = 0.869, *p* = 0.001, OR = 2.385 (95%CI 1.399–4.069)	B = −0.062, *p* = 0.894, OR = 0.940 (95%CI 0.375–2.355)	B = 0.726, *p* = 0.081, OR = 2.066 (95%CI 0.914–4.672)	B = 0.555, *p* = 0.046, OR = 1.742 (95%CI 1.011–3.002)
Residence	B = −0.187, *p* = 0.004, OR = 0.829 (95%CI 0.729–0.943)	B = 0.041, *p* = 0.727, OR = 1.042 (95%CI 0.826–1.316)	B = −0.264, *p* = 0.010, OR = 0.768 (95%CI 0.629–0.938)	B = −0.058, *p* = 0.391, OR = 0.943 (95%CI 0.826–1.078)
Education	B = −0.024, *p* = 0.866, OR = 0.976 (95%CI 0.738–1.291)	B = 0.069, *p* = 0.786, OR = 1.071 (95%CI 0.651–1.762)	B = 0.028, *p* = 0.895, OR = 1.029 (95%CI 0.674–1.570)	B = −0.016, *p* = 0.914, OR = 0.984 (95%CI 0.736–1.317)
	Physical violence			
	Before the pandemic	Change		During the pandemic
		In the direction of growth	In the direction of decline	
Model	*p* = 0.516	*p* = 0.778		*p* = 0.427
Intercept	B = −2.579, *p* < 0.001	B = −3.447, *p* < 0.001	B = −3.362, *p* < 0.001	B = −2.523, *p* < 0.001
Gender	B = −0.415, *p* = 0.156, OR = 0.660 (95%CI 0.372–1.172)	B = −0.005, *p* = 0.994, OR = 0.995 (95%CI 0.289–3.425)	B = −0.302, *p* = 0.397, OR = 0.739 (95%CI 0.367–1.487)	B = −0.376, *p* = 0.337, OR = 0.687 (95%CI 0.319–1.478)
Age	B = 0.470, *p* = 0.236, OR = 1.600 (95%CI 0.736–3.477)	B = −0.459, *p* = 0.628, OR = 0.632 (95%CI 0.098–4.055)	B = 0.674, *p* = 0.166, OR = 1.962 (95%CI 0.755–5.094)	B = −0.128, *p* = 0.817, OR = 0.880 (95%CI 0.298–2.598)
Residence	B = −0.006, *p* = 0.951, OR = 0.994 (95%CI 0.821–1.204)	B = −0.133, *p* = 0.521, OR = 0.875 (95%CI 0.582–1.316)	B = 0.031, *p* = 0.794, OR = 1.032 (95%CI 0.816–1.305)	B = −0.103, *p* = 0.426, OR = 0.902 (95%CI 0.701–1.162)
Education	B = −0.185, *p* = 0.439, OR = 0.832 (95%CI 0.521–1.326)	B = −0.331, *p* = 0.642, OR = 0.718 (95%CI 0.178–2.904)	B = −0.147, *p* = 0.589, OR = 0.863 (95%CI 0.506–1.471)	B = −0.297, *p* = 0.452, OR = 0.743 (95%CI 0.343–1.610)

## Data Availability

Data will be made available upon request. The data are not publicly available due to privacy and ethical border restrictions in our country.

## Supplementary Materials

The following supporting information can be downloaded at: https://www.mdpi.com/article/10.3390/brainsci14010024/s1, Table S1: Detailed baseline characteristics.
