# Supplementary material for: Evaluation of Psychological and Physical Violence towards Children and Adolescents before and during the COVID-19 Pandemic in the Lodz Voivodship
Source: Brain Sci. 2023 Dec 25;14(1):24. doi: 10.3390/brainsci14010024 (PMC10813170; doi:10.3390/brainsci14010024)
Supplement: Supplementary file 1 [file brainsci-14-00024-s001.zip › brainsci-2784778-supplementary.pdf]

Supplementary Table S1. Detailed baseline characteristics

		Whole study group	Psychological violence		Physical violence	
			Before the pandemic	During the pandemic	Before the pandemic	During the pandemic
**Whole study group**		**782**	131 (16.8%)	115 (14.7%)	50 (6.4%)	27 (3.5%)
Age (years)		14.53±2.60	15.46 ±1.97	15.24 ±1.98	14.82 ±2.08	13.93 ±2.50
Age	Under 15	317 (40.5%)	31 (9.8%)	33 (10.4%)	18 (5.7%)	14 (4.4%)
	15 and above	465 (59.5%)	100 (21.5%)	82 (17.6%)	32 (6.9%)	13 (2.8%)
Gender	Female	293 (37.5%)	34 (11.6%)	31 (10.6%)	23 (7.8%)	13 (4.4%)
	Male	486 (62.1%)	96 (19.8%)	83 (17.1%)	27 (5.6%)	14 (2.9%)
	Non-binary	3 (0.4%)	1 (33.3%)	1 (33.3%)	0 (0.0%)	0 (0.0%)
Pandemic wave	2nd	480 (61.4%)	79 (16.5%)	70 (14.6%)	26 (5.4%)	15 (3.1%)
	4th	302 (38.6%)	52 (17.2%)	45 (14.9%)	24 (7.9%)	12 (4.0%)
Residence	City of more than 100 thousand	64 (8.2%)	15 (23.4%)	7 (10.9%)	5 (7.8%)	2 (3.1%)
	City of 20-100 thousand	241 (30.8%)	54 (22.4%)	44 (18.3%)	16 (6.6%)	11 (4.6%)
	Town of 10-20 thousand	84 (10.7%)	10 (11.9%)	12 (14.3%)	4 (4.8%)	2 (2.4%)
	Town of less than 10,000	38 (4.9%)	3 (7.9%)	4 (10.5%)	2 (5.3%)	2 (5.3%)
	Village	355 (45.4%)	49 (13.8%)	48 (13.5%)	23 (6.5%)	10 (2.8%)
Building	Block of flats	197 (25.2%)	32 (16.2%)	26 (13.2%)	14 (7.1%)	7 (3.6%)
	Terraced house	39 (5.0%)	7 (17.9%)	8 (20.5%)	1 (2.6%)	0 (0.0%)
	Detached house	546 (69.8%)	92 (16.8%)	81 (14.8%)	35 (6.4%)	20 (3.7%)
Room	Own room	15 (1.9%)	4 (26.7%)	3 (20%)	2 (13.3%)	1 (6.7%)
	Shared with someone	111 (14.2%)	20 (18%)	16 (14.4%)	8 (7.2%)	4 (3.6%)
	no own	656 (83.9%)	107 (16.3%)	96 (14.6%)	40 (6.1%)	22 (3.4%)
Number of household members	2	49 (6.3%)	8 (16.3%)	7 (14.3%)	3 (6.1%)	2 (4.1%)
	3	185 (23.7%)	39 (21.1%)	30 (16.2%)	16 (8.6%)	12 (6.5%)
	4	317 (40.5%)	49 (15.5%)	45 (14.2%)	17 (5.4%)	9 (2.8%)
	5	145 (18.5%)	26 (17.9%)	25 (17.2%)	11 (7.6%)	4 (2.8%)
	6 and more	86 (11.0%)	9 (10.5%)	8 (9.3%)	3 (3.5%)	0 (0.%)
Siblings	0	154 (19.7%)	27 (17.5%)	18 (11.7%)	9 (5.8%)	3 (1.9%)
	1	431 (55.1%)	75 (17.4%)	65 (15.1%)	26 (6%)	15 (3.5%)
	2	142 (18.2%)	22 (15.4%)	22 (15.4%)	11 (7.7%)	7 (4.9%)
	3	38 (4.8%)	6 (15.8%)	7 (18.4%)	3 (7.9%)	1 (2.6%)
	4 and more	17 (2.2%)	1 (5.9%)	3 (17.6%)	1 (5.9%)	1 (5.9%)
Education step	Primary school	371 (47.4%)	43 (11.6%)	40 (10.8%)	24 (6.5%)	15 (4%)
	Secondary school	333 (42.6%)	74 (22.2%)	63 (18.9%)	22 (6.6%)	11 (3.3%)
	Profiled high school	19 (2.4%)	6 (31.6%)	4 (21.1%)	1 (5.3%)	1 (5.3%)
	Technical school	54 (6.9%)	7 (13%)	7 (13%)	2 (3.7%)	0 (0.0%)
	Vocational school	5 (0.7%)	2 (40.0%)	1 (20.0%)	1 (20.0%)	0 (0.0%)
Type of school lessons	Stationary	42 (5.4%)	3 (7.1%)	4 (9.5%)	5 (11.9%)	3 (7.1%)
	Remote	707 (90.4%)	121 (17.1%)	108 (15.3%)	42 (5.9%)	21 (3%)
	Hybrid	33 (4.2%)	7 (21.2%)	3 (9.1%)	3 (9.1%)	3 (9.1%)

The results for continuous variables are shown as mean ± standard deviation, while for categorical variables as a number of subjects (percentage of the group).
